# Development of broadly neutralizing antibodies in HIV-1 infected elite neutralizers

**DOI:** 10.1186/s12977-018-0443-0

**Published:** 2018-09-05

**Authors:** Elise Landais, Penny L. Moore

**Affiliations:** 1 International AIDS Vaccine Initiative Neutralizing Antibody Center, The Scripps Research Institute, La Jolla, CA 92037 USA; 2 Department of Immunology and Microbiology, The Scripps Research Institute, La Jolla, CA 92037 USA; 3 International AIDS Vaccine Initiative, New York, NY 10004 USA; 4 Centre for HIV and STIs, National Institute for Communicable Diseases of the National Health Laboratory Service, Johannesburg, South Africa; 5 Faculty of Health Sciences, University of the Witwatersrand, Johannesburg, South Africa; 6 Centre for the AIDS Programme of Research in South Africa (CAPRISA), University of KwaZulu-Natal, Durban, South Africa

## Abstract

Broadly neutralizing antibodies (bNAbs), able to prevent viral entry by diverse global viruses, are a major focus of HIV vaccine design, with data from animal studies confirming their ability to prevent HIV infection. However, traditional vaccine approaches have failed to elicit these types of antibodies. During chronic HIV infection, a subset of individuals develops bNAbs, some of which are extremely broad and potent. This review describes the immunological and virological factors leading to the development of bNAbs in such “elite neutralizers”. The features, targets and developmental pathways of bNAbs from their precursors have been defined through extraordinarily detailed within-donor studies. These have enabled the identification of epitope-specific commonalities in bNAb precursors, their intermediates and Env escape patterns, providing a template for vaccine discovery. The unusual features of bNAbs, such as high levels of somatic hypermutation, and precursors with unusually short or long antigen-binding loops, present significant challenges in vaccine design. However, the use of new technologies has led to the isolation of more than 200 bNAbs, including some with genetic profiles more representative of the normal immunoglobulin repertoire, suggesting alternate and shorter pathways to breadth. The insights from these studies have been harnessed for the development of optimized immunogens, novel vaccine regimens and improved delivery schedules, which are providing encouraging data that an HIV vaccine may soon be a realistic possibility.

## Background

The design of a preventative HIV vaccine is one of the major current public health challenges. Despite the global successes of antiretroviral therapy, rates of new infections, especially in sub-Saharan Africa, show little sign of abating, and indeed in some areas as many as half of young women are HIV infected [1]. Despite a massive effort, no vaccine has thus far been able to elicit protective neutralizing antibodies. However, extraordinary progress has been made in understanding the immune response to HIV infection and in defining viral targets. We now have a detailed understanding of the obstacles we face in eliciting protective antibodies, and this has enabled the design of new immunogens and vaccine strategies, many of which are based on studies of infection, and will enter clinical trials in the next months and years.

The major focus for HIV vaccine design is the elicitation of broadly neutralizing antibodies (bNAbs), capable of preventing entry by diverse viruses by binding to conserved regions on the HIV envelope glycoprotein trimer, which is the sole entry complex for HIV. This focus on bNAbs is based on the narrow window between HIV infection and the establishment of latency, ideally requiring antibodies to block viral entry. In contrast, although CTL responses have been shown to contribute to HIV control and slow disease progression [2–4], these responses are unlikely to protect from infection. Compelling evidence from animal studies shows that passive administration of bNAbs into non-human primates provides complete protection from mucosal challenge [5]. Non-broadly neutralizing antibodies, though capable of Fc effector functions such as antibody dependent cellular cytotoxicity, do not protect as well [6–8], further supporting a focus on neutralization. These findings, which are currently being further tested in the first human clinical trials of bNAbs as prophylaxis, suggest that such antibodies, if elicited at sufficiently high titers by vaccination, would be protective.

However, eliciting such bNAbs is fraught with difficulties. The Env protein, which consists of three gp120 and three gp41 molecules, has formidable defenses that hinder bNAb development [9]. The trimer is conformationally dynamic, extremely sequence variable, particularly in the antibody accessible regions of the envelope, sparsely arrayed on viral particles [10] and massively glycosylated, with glycans so tightly packed that they occlude much of the underlying protein surface [11]. Immunological decoys in the form of non-functional envelope proteins such as gp41 stumps, monomeric forms of gp120 and non-native protomers (such as uncleaved trimers) that expose non-neutralizing epitopes normally buried in the trimer, add a further layer of complexity [12].

Despite these barriers, infected individuals mount a vigorous neutralizing response though these initial responses are almost entirely strain-specific, targeting highly variable regions of Env [13–17]. However, over 2–3 years of infection, many people develop some degree of cross-reactivity [18], and a small subset of HIV infected individuals mount extremely potent and broad responses. These “elite neutralizers”, which are the focus of this review, have been the subject of intense study, in the hope that they provide a template for HIV vaccine design. Extensive functional (binding and neutralization) and structural (electron microscope, crystallography and glycobiology) characterization of these antibodies, and their precursors, has shed light on the complex molecular mechanisms by which they achieved breadth. Our increased understanding of the unusual features of bNAbs, and the failure of traditional vaccine strategies, have led the field to consider next-generation vaccine regimens which are based on the deep understanding of the host and viral factors leading the development of such antibodies. In this review, we provide a summary of these studies, and define some gaps that need to be addressed to develop an effective HIV vaccine.

## HIV targets and how these are accessed by bNAbs

**Fig. 1:**
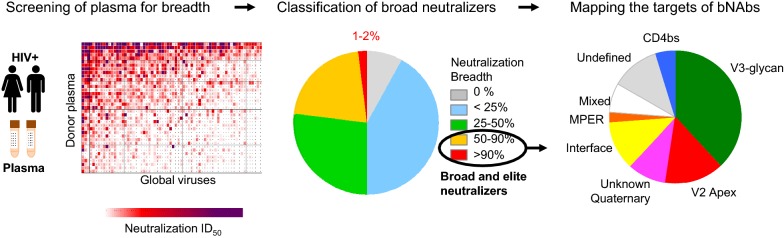
Identification of HIV-1 elite neutralizers and epitope mapping. Typically, plasma samples collected from HIV-1 infected individuals are tested for neutralization against panels of global env-pseudotyped viruses. Volunteers are ranked based on a their neutralization breadth and potency. The broad neutralizing activity in the top neutralizers is then mapped for epitope specificity using mutant viruses, and peptide and protein adsorptions. Reproduced with permission from [21]

One of the major reasons to study elite neutralizers has been the opportunity to define the targets of bNAbs on the HIV envelope. Epitope mapping was initially conducted using polyclonal plasma [19–21] (Fig. 1), but the development of new technologies has enabled efficient isolation of monoclonal antibodies, allowing much finer mapping of epitopes. Hundreds of bNAbs have now been isolated by amplification, sequencing and cloning of immunoglobulin gene transcripts from single B-cells identified by functional screening of micro-cultures and/or antigen-specific sorting. These data show that much of the Env can be targeted by bNAbs, with six distinct target regions identified on the HIV-1 envelope, almost all of which involve glycans. These are the V2-glycan site, the V3-glycan super-epitope, the membrane proximal external region (MPER), the CD4 binding site (CD4bs) and the gp120-gp41 interface, including the fusion peptide [22–24]. Most recently, antibodies targeting the so-called “silent face” have completed coverage of the Env glycoprotein [25]. However, plasma mapping studies suggest that further epitopes or sub-epitopes may remain to be identified. Novel approaches which do not rely on our knowledge of existing epitopes, such as the recently described use of cryo-electron microscopy of antibody-trimer complexes to map the specificities of plasma responses [26], will be informative in defining additional targets and designing more specific baits for B cell isolation.

For some bNAb epitopes, there is a degree of promiscuity with which an epitope can be recognized [9, 27]. For example, the V3-glycan supersite is relatively accessible to antibodies, perhaps explaining the prevalence of these bNAbs in infection (Fig. 1). BNAbs to this epitope show variable angles of approach centered around a series of conserved glycans at N301 and N332, but incorporating more variable elements in V1, V3 and V4 [28–31]. Similarly, bNAbs targeting the gp120/gp41 interface have diverse footprints delineating different sub-epitopes [22, 32–35]. However, other epitopes can only be accessed through very constrained angles of approach, forcing the immune system to utilize unusual structural features to access these. The stringent requirements for accessing these epitopes are reflected in the features of bNAb precursors, many of which have unusual features that are rare in the human immunoglobulin repertoire. The best examples of this are the VRC01-like CD4bs bNAbs that are characterized by conserved genetic and structural features which enable a common angle of approach [36–38]. This includes short antigen binding loops, required to avoid steric clashes with hypervariable regions and glycans [39]. In contrast, V2-directed bNAbs require a long anionic CDRH3 to penetrate the glycans protecting the apex of the trimer [40–43]. MPER bNAbs also use long variable loops and often develop membrane binding in order to access their epitopes [44, 45]. However, long loops and hydrophobic surfaces are associated with autoreactivity, such that these precursors are frequently deleted through tolerance mechanisms [43, 46–52]. Defining commonalities among bNAb precursors is the basis of “germline targeting” vaccine strategies, that are being pursued by several groups [39–41, 53–56]. Ongoing studies of immunoglobulin repertoires in diverse populations will provide insights into the possibility of reliably eliciting such bNAbs.

One feature that is common to bNAbs to several epitopes is an unusually high level of somatic hypermutation (SHM). Mutations are acquired in the complementarity determining regions of the antibodies which generally form the paratope, but also in the framework regions of antibodies which are normally more conserved [57]. Although the role of these mutations in conferring breadth is evident in studies of their ontogeny from precursors that are strain-specific [47, 58, 59], many of these mutations are “neutral”, conferring no benefit in terms of breadth, and simply a consequence of prolonged maturation in the context of chronic infection [60, 61]. The isolation of less mutated antibodies, described below, may therefore fill an important gap in the field. Much SHM is associated with the need to accommodate the Env glycan shield, either through direct contact and/or by avoiding glycans. Glycan adaptation often involves insertion/deletion events [29] which are rarely observed in the B-cell repertoire as they are less likely to be productive. Recognition of glycans is itself a limitation, as glycans are typically tolerogenic and are considered “self” epitopes by the immune system. Although some degree of poly- or autoreactivity has been reported for CD4bs (PCIN63, VRC01, 12A21) and to a lesser extent, V3-glycan directed bNAb lineages, this is particularly associated with MPER bNAbs (4E10, 2F5 DH511) [62]. Notably, engineering bNAbs to achieve enhanced breadth and potency sometimes results in enhanced polyreactivity, suggesting that maturation towards breadth is balanced by the need to avoid polyreactivity in the maturation of these lineages [63, 64]. Together, these features suggest complex developmental pathways that pose challenges to traditional vaccine strategies.

## Factors associated with the development of breadth

Longitudinal studies of the kinetics of plasma breadth have shown that bNAbs develop incrementally, often taking 2–3 years to emerge [19, 21]. This prolonged process suggests that extensive evolution of antibody responses is needed. Indeed, breadth has been associated with high viral loads, duration of infection, viral diversity and low CD4 T cell counts [19, 21, 65–68]. Furthermore, high overall plasma IgG levels and anti-Env IgG binding titers correlate with breadth, suggesting donors with breadth may access a more diverse repertoire of anti-Env Ab responses [21]. These findings emphasize the high levels of antigenic stimulation required to drive the extensive SHM often seen in bNAbs. However, there is also evidence that more specific viral attributes contribute to the development of breadth, with infection with subtype C viruses associated with enhanced breadth, and a bias to V2-glycan directed responses compared to the CD4bs responses more commonly observed in subtype B infected individuals [21, 65]. Another key factor associated with bNAb development is the level of circulating T follicular helper cells and germinal center (GC) function, which likely supports the SHM required for continued maturation [69, 70]. Recent data also showed that HIV-specific Fc effector function early in infection predicts the development of bNAbs, suggesting that intrinsic immune factors within the GC provide a mechanistic link between the Fc and Fab of HIV-specific antibodies [71]. Conversely, low levels of T regulatory cells, possibly enabling survival of B-cell intermediates with potential for autoreactivity, were also associated with development of bNAbs [72].

Superinfection has also been associated with broader antibody responses in some cohorts [73, 74], but not others [75]. This is an appealing observation for vaccine design, suggesting the possibility that superinfection boosts responses primed by the initial infecting virus, analogous to heterologous prime boost vaccines. However, a detailed comparison of the kinetics and targets of plasma antibodies in four superinfected donors suggested that superinfection was associated with de novo responses to both viral variants, and did not drive neutralization breadth (Sheward, Moore and Williamson, in press). This is further supported by the isolation of monoclonal antibodies (mAbs) from two superinfected donors, CAP256 and QA013. In CAP256, who developed extraordinarily potent bNAbs [76], these were directed only at the superinfecting virus [47]. Similarly, mAbs isolated from donor QA013 neutralized either primary infecting or superinfecting viruses, with none cross-neutralizing both. Furthermore, bNAbs in QA013 were largely attributable to mAbs targeting the superinfecting virus, with the mAbs that arose to the primary infecting virus only making a minor contribution to plasma breadth [77]. This suggests that HIV superinfection may enhance breadth through additive responses to each individual virus, consistent with the small effect seen in cohort studies [73], rather than through the boosting of memory responses, a distinction that is important for HIV vaccine design.

## Pediatric bNAb donors: a unique group of elite neutralizers

A group particularly interesting for HIV vaccine design is pediatric donors who appear to be enriched for “elite neutralizers”. BNAbs in HIV-infected children arise early in the course of infection, often during the first 2 years of life, and become more broad and potent than in adults, with 70% of children developing bNAbs that are equivalent to the top 20% of adults [78–80]. Chronically infected children frequently have an unusual phenotype of consistently high viral loads but normal CD4 counts, which may be highly conducive to the development of breadth [78]. Mapping studies show that bNAbs in children largely target previously defined epitopes, including the V2-glycan, V3-glycan, CD4bs and gp120-gp41 interface [81]. Remarkably, however, three quarters of children had antibodies targeting as many as four distinct bNAb epitopes with breadth mediated by a combination of these specificities. This polyclonality, which is also sometimes seen in adults [20, 21, 82–85], may be more pronounced in chronically infected children due to persistently high viral loads in this group, which is strongly linked to the development of neutralization breadth in adults [21, 65, 86–88]. The extraordinary breadth in these donors may suggest fundamental differences in their development (described in more detail below). This is supported by the isolation of mAb BF520.1 that targets the V3-glycan, like many adult bNAbs, but does so despite low levels of SHM, and in the absence of insertions/deletions that characterise bNAbs to this epitope [80]. The polyclonal nature of pediatric bNAbs may suggest that the immunoglobulin repertoire in children is more diverse than that of adults, or that maternal antibodies may shape the maturation of bNAbs, as has been observed in passive antibody administration in adults [89, 90]. Lastly, studies of GCs in children will shed light into whether these are functionally distinct from those of adults. Several recent studies suggest that children may be fundamentally better at generating antibody responses in vaccination and infection [78, 79, 91–94], which may be valuable for HIV vaccine design. Additional studies will therefore be important in providing insights into whether HIV infection, and therefore vaccination, may induce unique antibody responses in pediatric donors.

## Deciphering molecular pathways towards neutralization breadth

**Fig. 2:**
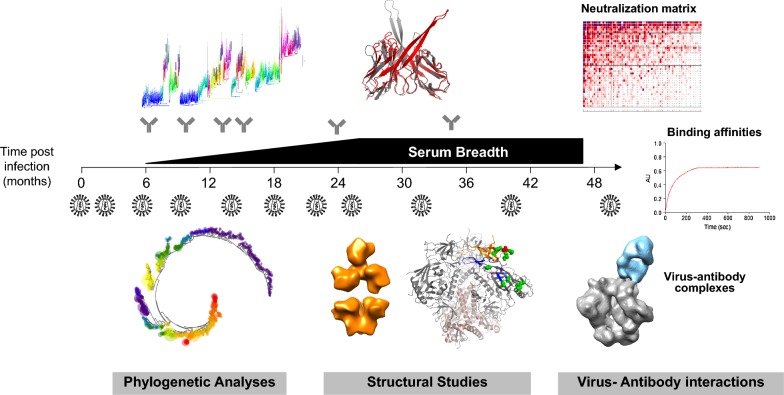
HIV and bNAb co-evolution studies. Longitudinal PBMCs samples are used for bNAb isolation (by functional screening of single B-cell micro-culture and/or antigen-specific cell sorting) and NGS sequencing of the memory B-cell repertoire. In parallel, corresponding longitudinal plasma samples are used to sequence and clone viral *env* variants. NGS data are used to re-construct the Ab and Env phylogenies over the course of infection. Cloned bNAbs and Env variants are functionally and structurally evaluated, both individually and in complex, to retrace the evolution of the virus-antibody interaction from elicitation to acquisition of neutralization breadth and inform vaccine design

A major focus has been the need to define the cellular and molecular mechanisms leading to the development of bNAbs. The “rational design” vaccine approach critically relies on the identification and characterization of B-cell precursors and key relevant Ab intermediates as well as the Env variants responsible for the elicitation and maturation of these bNAbs. Recent advances in next-generation sequencing (NGS) technologies have been key for such studies, allowing unprecedented analysis of the memory B-cell repertoire and of the viral envelope diversity within individuals [95]. This has enabled comprehensive, multidimensional studies deciphering the molecular interplay between the virus and B-cell response over the course of infection (Fig. 2).

To date, bNAb maturation has been studied for 14 lineages isolated from 13 HIV-infected donors (Table 1). The developmental pathways of very potent and broad CD4bs (VRC01, N49P7, N60P25.1) and MPER (10E8 and DH511) bNAbs were evaluated using chronic samples from four donors and provided key insights into their maturation. In addition, longitudinal sampling from HIV-infected donors enabled seven virus antibody co-evolution studies for bNAb lineages targeting the CD4bs (CH103 and CH235 from donor CH505, and PCIN63 from donor PC63), the V2 apex (CAP256-VRC26 from donor CAP256 and PCT64 from donor PC64) and the V3-glycan high mannose patch (PCDN from donor PC76, DH270 from donor CH848 and PCIN39 from donor PC39). Such studies of the longitudinal development of bNAbs were a unique opportunity, especially as clinical guidelines have moved towards early treatment of HIV infection, and were extremely valuable for the HIV vaccine effort. These longitudinal mAb studies built on previous plasma studies [19, 66] to better define the timing of breadth and identified bNAb precursors between 3 and 16 months post-infection, with maturation towards breadth taking 1–2 years, depending on the level of breadth and associated SHM achieved by the lineage.

**Table 1:** Studies of the maturation of broadly neutralizing antibodies

Epitope	Donor	Sampling	Ab lineage	Breadth Median IC50	VH-gene SHM (%nt)	VL-gene	CDR3 length	Indels	Poly/autoreactivity	Viral subtype	Viral sequencing	UCA binds/neutralizes T/F	References
CD4bs	NIH45	Chronic	VRC01	89%; 0.3 µg/mL	VH1-2 32%	VK3-20	H: 14 aa L: 5 aa	Yes Del CDRL1	+	B	No		[36, 38]
	DRVI01	Chronic	DRVIA7	< 10%	VH1-2 19%	VK1-5	H: 13 aa L: 5 aa	No		B	Yes		[120]
	CH505	Longitudinal	CH103	55%; 4.54 µg/mL	VH4-59 14%	VL3-1	H: 15 aa L: 10 aa	Yes Del CDRL1	++	C	Yes	Neutralization	[58]
	CH505	Longitudinal	CH235	90%; 0.6 µg/mL	VH1-46 28%	VK3-15	H: 15 aa L: 8 aa	No	++	C	Yes	Weak binding	[108, 119]
	N60	Chronic	N60P25.1	73%	VH1-2 33%	VK1-5	H: 13aa L: 5 aa	No		B	No		[125]
	N49	Chronic	N49P7	98%; 0.1 µg/mL	VH1-2 33%	VL2-11	H: 19aa L: 5 aa	Yes Del CDRL1		B	No		[125]
	PC063	Longitudinal	PCIN63	80%; 0.24 µg/mL	VH1-2 15%	VK1-5	H: 15 aa L: 5 aa	No	Variable	C	Yes	No binding	Landais et al. (unpublished)
V2-glycan	CAP256	Longitudinal	VRC26-CAP256	63%; 0.003 µg/mL	VH3-30 12%	VL1-51	H: 36 aa L: 13 aa	No	–	C	Yes	Neutralization	[47, 96]
	PC064	Longitudinal	PCT64	37%; 0.42 µg/mL	VH3-15 13%	VK3-20	H: 25aa L: 8 aa	No	±	A	Yes	Binding of 293S-expressed Env	[97, 98] .
V3-glycan	PC076	Longitudinal	PCDN	47%; 0.53 µg/mL	VH4-34 16%	VK3-20	H: 22 aa L: 8aa	No	±	C	Yes	No binding	[59]
	PC039	Longitudinal	PCN39	45%; 0.03 µg/mL	VH4-34 16%	VK3-20	H: 22 aa L: 10aa	Yes Ins CDRH1	++	C	Yes	No binding	Murrell, Landais et al. (unpublished)
	BF520	Longitudinal (Infant)	BF520.1	58%; 1.95 µg/mL	VH1-2 7%	VK3-15	H: 20 aa L: 7aa	No		A	Yes	Cell surface binding	[80]
	CH848	Longitudinal	DH270	55%; 0.08 µg/mL	VH1-2 13%	VL2-23	H: 20aa L: 9 aa	No	±	C	Yes	Weak binding	[102]
MPER	N152	Chronic	10E8	98%; 0.35 µg/mL	VH3-15 21%	VL3-19	H: 22 aa L: 11 aa	No	–	B	No		[149]
	CH0210	Chronic	DH511	99%; 1.04 µg/mL	VH3-15 18%	VK1-39	H: 22 aa L: 11 aa	No	++	C	No		[150]

A key aspect for vaccine design has been the use of longitudinal memory B-cell NGS to accurately infer the sequence of the unmutated common ancestor (UCA) for several bNAb lineages. The accuracy of this inference is highly dependent on the availability of early, less mutated bnAb lineage sequences. In some cases, these UCAs can bind (CH235, CH103, PCT64) and even neutralize early Env variants (CH103, CAP256-VRC26 and PCT64) [47, 58, 96–98]. However, in others, no binding of precursor B-cells was detectable (PCDN, PCIN39, DH270, PCIN63). This observation led to the hypothesis that some of these bNAbs may have matured from responses to other pathogens, however it is also possible that affinity undetectable in existing assays might have been sufficient to induce BCR signaling and initiate clonal expansion in vivo [99]. Moreover, this low affinity, mainly due to the sub-optimal epitope presentation on the Env protein, can be overcome by synthetic minimal epitope molecules such as CD4bs mimics eOD-GT8 and 426c (VRC01) [54, 100, 101], short V3-glycopeptides (DH270) [102, 103] and V2-apex scaffolds [40, 41], providing opportunities for immunogen design.

Activation of the naïve B-cell precursor induces a rapid expansion and diversification of the mAb lineage, which is typically followed by a contraction phase that is likely due to rapid viral escape preventing further maturation [59]. Memory B-cell repertoire analyses have revealed subsequent multi-limb maturation as the early antibody intermediates undergo different fates, depending on whether they can still recognize emerging new viral variants [47, 59]. Some branches display limited maturation (“dead-end” sub-lineages) due to a failure to recognize emerging viral variants [96]. Other branches continue to accumulate SHM in distinct parallel pathways through continual adaptation to new variants in the autologous virus population [47, 59, 96]. However, continued maturation is not always associated with neutralization breadth. Bhiman et al. [96] described highly mutated “off-track” CAP256-VRC26 mAbs, which have more than 20% SHM but limited neutralization breadth. Similar observations have been made in other bNAb lineages [58, 104, 105], and as mAb isolation methodologies are specifically designed to recover bNAbs, the proportion of bNAb lineages that are off-track is unknown.

Structural studies carried out in parallel to the repertoire analyses have also provided critical insights into the molecular basis of affinity maturation (reviewed in [106]). The detailed molecular mechanisms allowing neutralization breadth via epitope focusing and by adaptation to the glycan shield (through direct contact or by reducing steric clashes) have been better defined, and exploited for immunogen design (see below). Overall, the low affinity of bNAb precursors for cognate antigens is associated with lower thermodynamic stability, particularly of CDR loops, and SHM leads to epitope focusing [107], improved shape complementarity and increased buried surface area at the interface with the antigen, by conformational re-organization and stabilization of the paratope [47, 97]. Within bNAb lineages, these changes can occur independently within different sublineages maturing to acquire breadth [46, 47, 58, 96, 104, 105]. These varying pathways to breadth strongly demonstrate the plasticity of the immune system, which, even within one antibody lineage, can find multiple structural and genetic solutions to the same immunological problem.

## The role of viral variants in selecting bNAbs

These multiple pathways to bNAb maturation also highlight the power of the selective pressure exerted by emerging Env variants. A common finding from co-evolution studies is that of Env diversity as a key driver of breadth, with the emergence of cross-neutralization within bNAb lineages associated with a burst of viral diversity [47, 58, 97]. This early viral diversification is the result of selective pressure by early (strain-specific) precursors within the bNAb lineage [96], or by unrelated “helper” or “cooperating” neutralizing antibodies that target an overlapping epitope and drive viral mutations within the bNAb epitope [102, 108–110]. Maturing bNAb lineages are thus constantly exposed to a large variety of Env “immunotypes”, varying not only in amino acid sequence [96], but also in conformation and glycosylation profile [97]. These viral mutants are recognized by bNAb intermediates capable of tolerating emerging “immunotypes” within epitopes, and these continue to be selected during the next round of affinity maturation [96]. This constant exposure of the immune system to viral variants and glycoform heterogeneity selects those antibody sub-lineages able to tolerate viral diversity and indirectly drives the maturation of bNAbs [96]. The rate of viral escape also seems to be key. Within the CAP256-VRC26 lineage, constrained virus escape was associated with reduced infectivity and altered entry kinetics [111]. This observation is consistent with the likelihood that bNAb epitopes are conserved for functional reasons, with escape likely requiring compensatory mutations [97, 110], and suggests that slower viral escape supports the development of bNAbs through prolonged exposure to antigen [96, 97, 110, 111]. This hypothesis is further supported by the demonstration in vaccine studies that extended antigen availability enhances germinal center activity and resulting neutralizing Ab responses [112, 113].

BNAb development thus depends on a sustained feedback loop, in which Env diversity and high antigen levels lead to increased diversity in the Ab response. This i) increases the chances of stimulating relevant precursors, and ii) results in maturing bNAb lineages being exposed to multiple related antigens, generated as the virus explores multiple escape pathways within a restricted landscape, eventually leading to neutralization breadth. This supports a strategy of using sequential Envs from elite neutralizers as templates for immunogen design, which has recently shown promise in animal vaccination studies, albeit in the artificial context of mice engineered to express bNAb precursors [114].

## Can common patterns in bNAb development be exploited for immunogen design?

Although co-evolutionary studies provide fascinating insights into the immune system, their utility as models for vaccine design is based on the notion that there are inter-donor commonalities amenable to vaccine design. As more studies emerge, such common patterns are becoming evident, both in terms of antibody precursors and the viral variants that select them.

The exquisite structural homogeneity with which VRC01-class bNAb precursors bind to the HIV Env CD4bs has been exploited in the design of germline-targeting immunogens (eOD-GT8 and 426c) [54, 100, 101]. These could elicit narrowly neutralizing antibody responses with VRC01-like characteristics, namely VH1-2 gene usage, a 5 amino acid CDRL3 and a short/flexible CDRL1, in mouse models [53, 115–117]. As additional studies of V2 and V3-glycan directed bNAbs define common features in their precursors and in the viral envelopes bound by them, germline targeting is being expanded to other epitopes [40, 41, 55, 114, 118].

Similarly, comparison of the viral diversification that leads to breadth is identifying convergent envelope features, which could be harnessed for vaccine design. For example, in two V2-glycan bNAb donors, there is striking similarity in the viral variants associated with maturation of breadth [96, 97]. In both these donors, localized diversity at the same key V2 epitope residues, specifically residues 166 and 169, selected SHM that drove bNAb maturation toward breadth [96, 97]. Similarly, studies of CD4bs directed antibodies in several donors have highlighted the role of variation in loop D and in the V5 glycans in shaping breadth [58, 108, 119], whereas for V3-glycan bNAbs, shifting glycans at positions 332, 335 and 301, and mutations within the V3 GDIR motif are common escape mechanisms between donors [59, 102]. While these studies support the idea of rational vaccine design and provide the first real hope that common pathways may exist, systematic structural and functional evaluation of longitudinal bNAb/Env autologous complexes and a deeper understanding of Env conformational plasticity, will provide insight into how these interactions drive bNAb lineage maturation towards breadth.

## Are the unusual features of bNAbs an insurmountable hurdle for vaccine design?

The high level of SHM typically exhibited by bNAbs is seen as a major hurdle for vaccination. This is especially true of bNAbs targeting the CD4bs. Indeed, despite the detection of VRC01-class Ab precursors in the naïve repertoire of a majority of healthy individuals [101], bNAbs directed to the CD4bs rarely develop in infected individuals and typically only after several (> 5) years of infection. Although about two-thirds of the SHM seen in CD4bs directed bNAb VRC01 conferred no benefit in terms of neutralization breadth [60], adaptation to the glycan surrounding the CD4bs, such as position N276, appears to be a major hurdle for CD4bs VH1-2 antibody lineages to overcome. This was clearly demonstrated through the study of the DRIVA7 lineage which, despite being encoded by a VH1-2*02 gene, encoding the critical 5 amino acid CDRL3 associated with VRC01-class bNAbs, and acquiring critical mutations in the CDRH1, CDRH3 and FRH3, failed to achieve the light chain maturation necessary to adapt to HIV Env loop D and V5 glycans [120].

There are however some examples that suggest that there may be shortcuts to the development of breadth, providing a more feasible template for vaccine design. One such example is the CD4bs lineage from donor PC63, which has equivalent breadth and potency to VRC01, but substantially less SHM (13.7% compared to 31.6% of VH1-2 nucleotides) [121]. Notably, the SHM that appears in this lineage is highly focused at positions previously shown to be key CD4bs epitope contacts or glycan adaptation residues, suggesting that the viral variants in this donor might represent an ideal series of immunogens to drive the inclusion of “useful” SHM in CD4bs lineages, and avoiding the selection of redundant mutations that do not contribute to breadth.

In addition to the pediatric V3 glycan mAb BF520.1 described above [77], lower levels of SHM have also been described for adult bNAbs targeting the V3-glycan supersite. These include the PCDN [59], DH270 [102] and PCIN39 [122] bNAb lineages, further suggesting that shorter maturation pathways more compatible with vaccination can be achieved for this epitope. Furthermore, three of these V3-glycan lineages also achieved breadth without insertion/deletions (indels) in CDR loops, seen as another major roadblock for vaccination. Interestingly, in the fourth example, CDRH1 insertions of different lengths occurred independently in several branches of the PCIN39 lineage. Together, these studies suggest (i) that indels may not necessarily be required for V3-glycan bNAbs (ii) that indels may be less rare than anticipated, and that given the right selection, Ab lineages with insertions could be elicited by vaccination. Thus, despite substantial genetic and structural heterogeneity, the V3-glycan supersite remains an attractive vaccine target.

For bNAbs to the V2-glycan epitope, the long CDRH3s characteristic of this class of bNAbs arise during naïve rearrangement rather than during maturation [47], and are extremely rare in the naïve repertoire [49]. Nonetheless, such specificities, once triggered can rapidly mature towards breadth with moderate levels of SHM [47]. Furthermore, V2-glycan bNAbs PCT64, VRC38 and CH01 bear slightly shorter CDRH3 loops of ~ 25 amino acids, which are much more common in the naïve repertoire. Although bNAbs with shorter CDRH3s showed significantly lower breadth than the PG9, PGDM1400 and CAP256-VRC26 bNAbs that have longer CDRH3s, this reduced breadth is potentially off-set in a vaccine scenario by higher precursor frequency [82, 123]. This balance between breadth and more “normal” genetic features of antibodies raises the question of whether the focus on elite neutralizers may have resulted in missed opportunities. With the exception of germline targeting immunogens, most vaccines aim to elicit polyclonal responses to multiple epitopes, a scenario similar to that of most HIV infected individuals, who develop some cross-reactivity [18], often targeting multiple epitopes [20, 21, 82–85]. It is therefore possible that a renewed focus on more “elicitable” antibodies, with features more representative of the overall repertoire (e.g. few insertions or deletions, lower levels of SHM and average length CDRH3 s) may be valuable in honing vaccine candidates.

## Can studies of failed bNAb lineages tell us what the roadblocks are?

In addition to studies of more moderate neutralizers, studies of failed bNAb lineages may also be informative. Although most individuals mount robust neutralizing responses, the low proportion of individuals who develop breadth, and the fact that in some broad neutralizers, bNAbs represent a minority of the overall response [124, 125] suggests that the roadblocks impeding the development of breadth are profound. Analysis of the developmental pathway of Ab lineages failing to develop breadth would also shed light on the molecular events limiting the acquisition of neutralization breadth [126]. Defining both favorable and unfavorable maturation pathways may also allow a better evaluation of the responses elicited by immunization to determine whether they are on the “right” track.

## Leveraging studies of infection for vaccine design: What are the gaps?

**Fig. 3:**
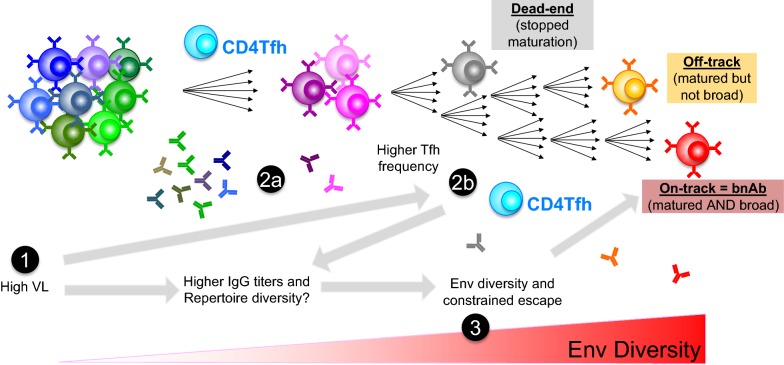
Factors associated with bNAb development, and how to leverage these factors in vaccination strategies. During infection, both the virus and the antibodies evolve rapidly through relatively random genetic variation. BNAb development is associated with factors likely increasing the chances of a productive encounter between rare low-affinity B-cells with unusual features, and rare Env variants with unusual characteristics. High antigen concentration (1), larger B cell repertoire (2a), antigen diversity (3), and increased germinal center activity (2b) are all factors that may be enhanced in vaccine design

Overall, the data reviewed here suggest a model based on the stochastic recruitment of rare, low-affinity precursor B cells, often with unusual features, by Env variants that may be equally unusual, followed by the survival of randomly generated intermediates with low affinity for emerging viral escape variants. Many of the factors associated with breadth (described above), effectively act by increasing the chances of successful bNAb generation. The main goal of vaccine design is to similarly increase the odds, by optimizing immunogens and adjuvants and by defining novel vaccine regimens and delivery schedules (Fig. 3 and Table 2).

**Table 2:** Translating insights from studies of infection into novel immunization strategies

		Mechanism in infection	Immunization
1	High viral load	Recruitment of larger B and T cell repertoires by increasing chances of activating lower affinity precursors due to higher antigen concentration	Germline-targeting immunogen design based on UCA features → high affinity prime Adjuvants to boost innate immune responses
2a	High anti-Env IgG Titers and helper lineages of neutralizing antibodies	More diverse repertoire increases chance of productive bNAb UCA encountering antigen May restrict/slow down viral escape	Adjuvants to ensure high Ab titers and posibly greater diversity Sustained delivery to ensure prolonged availability of Ags Adjuvants to boost innate immune responses
2b	High frequency of Tfh	May increase chances of activating lower affinity intermediates with unusual features, thereby sustaining affinity maturation and leading to higher SHM levels	Formulation should include T-cell epitopes Adjuvants to boost innate immune responses and GC reaction
3	Burst of viral diversity	Increased chances of activating/maturing low affinity intermediates with unusual features. Sustained affinity maturation and associated high levels of SHM	Immunogen design incorporating incremental diversity. Autologous versus heterologous boosts to avoid “dead-end” pathways Contemporaneous delivery of “variants” to quickly select relevant intermediates and positive selection

Numbering relates to Fig. 3

Several solutions are already being explored for the optimization of immunogens, such as the development of high-affinity germline targeting immunogens [39–41, 53–56, 103, 127]. Soluble stabilized Env trimers with key structural features are also a major focus in the field, to select critical antibody intermediates capable of binding the functionally relevant Env native conformation (reviewed in [128]). While the design of soluble trimers has greatly advanced the field, less is known about envelope signatures that impact the thermodynamics of natural viral trimers, perhaps affecting binding/neutralization by early bnAb intermediates. Understanding the stabilized conformation compared to membrane anchored spikes [98] and whether some degree of flexibility is necessary for productive interactions within developing bNAb lineages will be informative. Finally, variation in glycosylation also likely impacts trimer properties, and extending recent advances in glycobiology [11, 129, 130] will provide additional insights into controlling the glycan shield for acquisition of breadth [131, 132].

The rationale for further trimer stabilization is, in part, to reduce elicitation of “distracting”, mostly V3 directed, strain-specific responses, which might outcompete the bNAb lineages during affinity maturation [133]. However, during infection bNAbs develop in a highly competitive environment, and immunization studies showed that autologous neutralizing titers were not enhanced by reduction of V3 responses [113, 134]. Additionally, two elegant studies recently showed that most GCs retain substantial clonal diversity during affinity maturation of complex antigens [135, 136]. The effect of interclonal competition as a limiting factor for bNAb elicitation is thus not clear. Much of what we know about the GC reaction comes from studies mimicking an acute infection with non-variable model antigens, and B-cell affinity maturation and differentiation in chronic GCs are incompletely defined (Reviewed in [137–139]). Specifically, the accumulation of higher levels of SHM and its effect on self-reactivity [140], and the impact of antigenic drift associated with HIV infection on the formation of memory subsets need to be better understood. Ongoing studies aiming to directly sample GC B cells and T follicular helper and regulatory cells during infection, as has recently been done in immunization [112], will be important.

Translating viral studies from elite neutralizers to vaccine design is another active area of research. Though incorporating some antigen diversity seems crucial, vaccine regimens cannot match the Env diversity seen during infection. How much diversity is required is not known. Several studies suggest that sequential delivery of a mix of Env immunogens may be preferable over a repeated cocktail immunization for the elicitation of bNAb responses [141, 142]. This contrasts by GC modeling studies [143] and with findings from studies of elite neutralizers, where a burst of viral diversity typically precedes acquisition of breadth [47, 58, 59]. Furthermore, the association of bNAbs with slower viral escape, and the existence of antibody “dead-ends”, suggests that too much variation between prime and boost Envs may terminate a nascent lineage. While animal data suggests that a lineage of autologous Env may specifically drive the maturation of a particular lineage [55, 115, 144], whether this will be a generalizable response in vaccine recipients will require human trials.

A further challenge is how to drive maturing lineages to accommodate glycans, particularly where a given glycan, such as that at residue 276, is incompatible with binding with bNAb precursors, but globally conserved on viruses. One approach has been the creation of “glycan holes” in Env immunogens. These bare Env regions are immunodominant in vaccine studies [145, 146], however the potential of these neutralizing responses to mature to recognize or tolerate glycans is unclear. During infection, breadth is not determined by the overall autologous neutralizing response ([14, 19] Sheward, Moore and Williamson, in press). This highlights the remaining challenges in driving “glycan-hole” directed vaccine responses towards breadth. Finally, the need for sustained exposure to high concentrations of antigens, as in infected donors with high viral loads, has led to strategies incorporating subcutaneous and extended administration with promising improvements in nAb titers [113, 147, 148].

In summary, the identification of elite neutralizers, and the unprecedented detail in which these donors have been studied, has provided crucial immunological and virological insights into the development of bNAbs. The translation of these landmark studies into innovative vaccine strategies that seek to bypass the stringent limitations imposed by rare bNAb precursors, and drive these towards breadth is generating promising data in animal studies. Ongoing and planned experimental medicine trials in humans will take the HIV vaccine field into a hugely exciting era. For the first time in 30 years of an HIV pandemic that has devastated communities, these studies provide hope that an HIV vaccine may be achieved, but also provided immunological lessons that are being harnessed for the design of vaccines against other highly mutable, complex pathogens.

### Additional references in Table 1

[149, 150].
